# Garlic-Derived Metabolites Exert Antioxidant Activity, Modulate Gut Microbiota Composition and Limit *Citrobacter rodentium* Infection in Mice

**DOI:** 10.3390/antiox11102033

**Published:** 2022-10-15

**Authors:** Ling Zhu, Audrey I. S. Andersen-Civil, Josue L. Castro-Meija, Dennis S. Nielsen, Alexandra Blanchard, John E. Olsen, Stig M. Thamsborg, Andrew R. Williams

**Affiliations:** 1 Department of Veterinary and Animal Sciences, Faculty of Health and Medical Sciences, University of Copenhagen, 1870 Frederiksberg, Denmark; 2 Department of Food Science, University of Copenhagen, 1958 Frederiksberg, Denmark; 3 Pancosma|ADM, A One Business Center, La Piece 3, CH-1180 Rolle, Switzerland

**Keywords:** propyl propane thiosulfinate, propyl propane thiosulfonate, antioxidant activity, *Citrobacter rodentium*, nuclear factor erythroid factor 2-related factor 2

## Abstract

The garlic-derived compounds propyl propane thiosulfinate (PTS) and propyl propane thiosulfonate (PTSO) are metabolites with putative health benefits against intestinal inflammation that may be related to their antioxidant activity. However, the underlying mechanisms remain unclear, and whether PTS-PTSO can promote gut health by altering the microbiota and exert protection against enteric pathogens needs further investigation. Here, we explored the antioxidant activity of PTS-PTSO in murine macrophages in vitro, and in an in vivo model of bacterial infection with the bacterial pathogen *Citrobacter rodentium*. PTS-PTSO attenuated reactive oxygen species in lipopolysaccharide-stimulated macrophages in a nuclear factor erythroid factor 2-related factor 2 (Nrf2)-dependent manner, decreased nitric oxide levels both in macrophages in vitro and in the sera of mice fed PTS-PTSO, and had putatively beneficial effects on the commensal gut microbiota. Importantly, PTS-PTSO decreased faecal *C. rodentium* counts, concomitant with upregulation of Nrf2-related genes in colon tissue. Thus, PTS-PTSO mediates Nrf2-mediated antioxidant activity and modulates gut microbiota, which may protect the host against *C. rodentium* colonization. Our results provide further insight into how PTS-PTSO and related bioactive dietary compounds may reduce enteric infections.

## 1. Introduction

Enteropathogenic *Escherichia coli* (EPEC) are important human gut pathogens, which cause intestinal oxidative stress, severe diarrhea, and mortality, mainly in children. Severe cases may be treated with antimicrobials. However, the overuse of antibiotics not only causes drug-resistance, but also impairs normal gastrointestinal function, increasing the risk of metabolic disorders and inflammation [1]. Finding alternatives to current antibiotics is, therefore, a priority.

The murine enteric pathogen *Citrobacter rodentium* (*C. rodentium*) shares virulence mechanisms encoded from a pathogenicity island termed locus of enterocyte effacement (LEE) with EPEC, as well as enterohaemorrhagic *E. coli* (EHEH) and, like these *E. coli* types, it mainly affects the distal large intestine with low-level inflammation [2,3]. It has, therefore, been widely used as a model to study the pathogenicity of these pathotypes. Most studies indicate that host defenses against *C. rodentium* are connected to epithelial barrier function, the host gut microbiota and anti-inflammatory responses [3,4]. Typically, more reactive oxygen species (ROS) and pro-inflammatory cytokines such as IL-6, TNFα, and IL-17 are observed in *C. rodentium*-infected mice than in controls, together with decreased *Lactobacillus* spp. populations in the gut, suggesting that these factors can be potential targets, which may affect *C. rodentium* clearance [5]. Thus, compounds that modulate host oxidative stress, inflammatory responses, and/or the commensal microbiota, may be promising strategies for regulating resistance to infection.

Compounds from plants that are commonly found in dietary supplements have been reported to improve the outcome of intestinal diseases with a lower risk of drug-resistance, and have been reported to maintain the intestinal balance by regulating the commensal bacteria and gut immune responses, thereby promoting health and reducing disease [6]. Among the natural herbs, garlic (*Allium sativum L*.) has been used for thousands of years because of its medical effects, and its characteristic flavor and odor as a food additive [7]. It is reported that garlic can inhibit bacterial growth, including the growth of methicillin-resistant *Staphylococcus aureus*, *Helicobacter pylori* and *E. coli* [8,9]. Moreover, the anti-cancer, antifungal and immune-regulatory properties of garlic have also attracted attention. Major organosulfur compounds, such as allicin and alliin, are assumed to be responsible for these biological functions [10]. When garlic is crushed or processed, the enzyme alliinase will be released and this can react with Propiin (S-propyl-_L_-cysteine sulfoxide) to form propyl propane thiosulfinate (PTS) and propyl propane thiosulfonate (PTSO), two organosulfur compounds that are normally found together in high amounts in garlic extracts [11]. As secondary metabolites from garlic, PTS and PTSO exhibit potent antimicrobial effects against *Salmonella enterica*, *E.coli* and other enterobacteria [12]. Therefore, PTS and PTSO have been used both as a anti-bacterial material for industrial packaging and also as an dietary additive, where they may alleviate obesity-associated systemic inflammation, as well as improving intestinal microbiota homeostasis, indicating their potential use as novel treatments for inflammatory diseases and metabolic syndrome [13,14]. Thus, PTS-PTSO may influence EPEC and EHEC colonization in the gut through various mechanisms.

Recently, we demonstrated that PTS-PTSO has anti-inflammatory activity in murine macrophages in vitro, and attenuates inflammation caused by enteric helminth infection, altering the expression of genetic pathways involved in immune function and oxidative stress [15]. However, the mechanism(s) of antioxidant activities need to be further investigated, and the ability of these compounds to regulate the gut environment and protect enteric bacterial pathogens, including *C. rodentium* in mice, remains unclear. We hypothesized that PTS-PTSO exerts antioxidant properties that can modulate the gut environment to minimize inflammation and protect the host against enteric infection. Here, we show that PTS-PTSO reduced ROS and nitric oxide (NO) levels in lipopolysaccharide (LPS)-activated murine macrophages and regulated the murine gut microbiota (GM) composition. Moreover, PTS-PTSO reduced the faecal load of *C. rodentium* in experimentally challenged mice. Notably, PTS-PTSO enhanced the expression of antioxidant proteins related to Nrf2 signaling in both uninfected and infected mice. These results demonstrate that PTS-PTSO exerts strong Nrf2-mediated antioxidant signaling properties and can also protect the host against enteric infection, potentially through the antioxidant activity of PTS-PTSO. Thus, our data can aid in the design of dietary supplements based on garlic or related bioactive compounds to reinforce the robustness of the host to cope with intestinal challenges.

## 2. Materials and Methods

### 2.1. Garlic Metabolites

**Figure 1:**
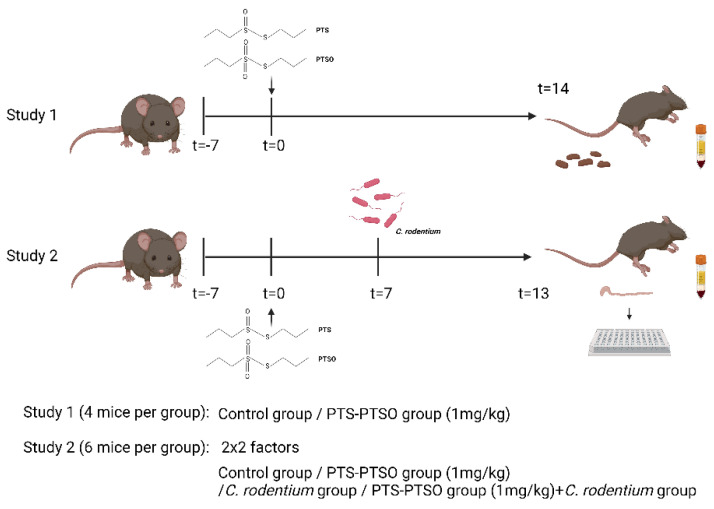
Design of the mouse studies.

The garlic product was provided by Pancosma SA (Rolle, Switzerland). It contains 40% PTS-PTSO (of which 80% consists of PTSO, and 20% PTS; Figure 1). The remainder of the extract solution consists of the solvent polysorbate-80. The stated experimental concentrations refer to final PTS-PTSO concentration used in assays or animal experiments. In all experiments, control cells and control mice were treated with an equivalent amount of polysorbate-80 as the PTS-PTSO-treated groups.

### 2.2. Cell Culture

RAW264.7 macrophages (ATCC-TIB-71) were cultured in DMEM medium (supplemented with 10% fetal bovine serum and 100 U/mL penicillin and 100 µg/mL streptomycin, all from Sigma-Aldrich). As previously described [16], RAW264.7 cells were split into 24-well plates for PTS-PTSO and LPS stimulation. After 2 h of incubation for appropriate cell attachment, 5 µM of the Nrf2 inhibitor ML385 (Sigma-Aldrich, Schnelldorf, Germany) was added for 2 h followed by 20 µM PTS-PTSO and 500 ng/mL LPS for 24 h stimulation, then ROS and NO production were measured.

### 2.3. Reactive Oxygen Species Assay

ROS production in RAW264.7 cells was measured using the fluorescent dye DCFH-DA (Sigma-Aldrich), as described previously [16]. In brief, cells were washed with serum-free DMEM medium, then 10 μM DCFH-DA was transferred onto the cells for 30 min incubation at 37 °C, 5% CO_2_. Next, cells were washed 2–3 times with serum-free medium, then detached with Accutase (Sigma-Aldrich) for ROS measurement by flow cytometry (Accuri C6, BD biosciences, San Jose, CA, USA).

### 2.4. Nitric Oxide Assay

NO accumulation in cells and serum was measured by the Griess reagent (Abcam, Cambridge, UK). Cell lysate or sera were prepared with cold nitrite assay buffer for 10 min on ice; then, the supernatant was collected after centrifuging at 10,000× *g* for 5 min. 100 μL Griess buffer was added to the supernatant for a 10 min incubation period, and the absorbance was measured at 540 nm.

### 2.5. Western Blot

Cells were washed twice with cold PBS (Sigma-Aldrich); then, RIPA buffer (ThermoFisher Scientific, Waltham, MA, USA) with Halt Protease Inhibitor was added onto the cells for lysis. The supernatant was collected by centrifuging at 10,000× *g* for 10 min. Protein in supernatant with loading buffer (Thermo Scientific) was separated on an iBolt 4–12% bis-tris gel (Invitrogen, Waltham, MA, USA), then transferred to nitrocellulose membrane using the iBlot 2 Transfer Stack and iBlot 2 Gel Transfer Device according to the manufacturer’s instructions. The total protein of each sample was quantified through REVERT™ Total Protein Stain kit (Li-COR, Lincoln, NE, USA) at 700nm. Subsequently, blocking was conducted by Intercept^®^ (TBS) Blocking Buffer (Li-COR, Lincoln, NE, USA) for 0.5 h–1 h, and the membrane was probed with rabbit anti-mouse PRDX1 (1:2000, Proteintech, Manchester, UK) and rabbit anti-mouse HO-1 (1:2000, Cell Signaling Technology, Frankfurt, Germany) at 4 °C, overnight. Then, the membrane was washed with TBST buffer (Li-COR, Lincoln, NE, USA), and incubated with secondary antibody (goat anti-rabbit, 1:30,000) for 1 h. The protein was detected and analyzed using LI-COR Odyssey^®^ Imagers (Li-COR, Lincoln, NE, USA) at 700 nm and 800 nm. Relative normalized protein was calculated according to REVERT™ Total Protein Stain Normalization protocol.

### 2.6. Animal Experiments

Mice experimentation was approved by the Experimental Animal Unit, University of Copenhagen, and conducted in line with the guidelines of the Danish Animal Experimentation Inspectorate (License number 2020-15-0201-00465). Female mice (C57BL/6 strain, 6–8 weeks of age; Enivgo, The Netherlands) were used and housed in individually ventilated cages with ad libitum access to mouse chow (DF-30, SAFE, Augy, France). Mice were administered PTS-PTSO in drinking water at a dosage of 1 mg/kg body weight, based on an intake of 4 mL water/mouse/day. Mice in the untreated groups received an equivalent amount of polysorbate 80 in the drinking water to the intervention groups. For infection studies, *C. rodentium* (strain DBS100; ATCC 51459) was grown overnight in LB broth, and mice were gavaged with 10^9^ CFU/mouse, with the dose confirmed retrospectively by serial dilution and plating on McConkey agar. Mice were sacrificed at the indicated timepoints by cervical dislocation, and faeces were collected from the colon for GM analysis (see below) or assessment of *C. rodentium* burden by homogenization of faeces in PBS before serial dilution and plating on McConkey agar overnight at 37 °C to determine CFU/g faeces. Colonic tissues were stored in RNAlater for RNA extraction, blood was collected prior to euthanasia and serum was harvested for analysis of NO or glutathione production (study design shown below in Figure 1).

### 2.7. Faecal Microbiota Analysis

The Bead-Beat Micro AX Gravity kit (A&A Biotechnology, Gdansk, Poland) was used for faecal DNA extraction in accordance with the manufacturer’s instructions. Additionally, lysozyme and mutanolysin were supplemented in lysis buffer for stronger bacterial cell wall degradation before DNA extraction. The 16S rRNA gene V3-region was amplified and sequenced as previously described [16,17]. The raw dataset containing pair-ended reads was merged and trimmed, and zero-radius operational taxonomic units (zOTU) obtained using the Greengenes (13.8) 16S rRNA gene collection as a reference database. Further analysis was conducted using the Quantitative Insight into Microbial Ecology open-source software package QIIME 2 (v2019.4.0). Taxonomical assignments were obtained using the EZtaxon for 16S rRNA gene database. For subsequent analyses within this dataset, samples were normalized to 7000 reads. Principal Coordinates Analysis (PCoA) was performed on Bray–Curtis distances and differences in treatments evaluated with analysis of similarities (ANOSIM).

### 2.8. Total Glutathione Assessment

Total glutathione levels of serum of mice were measured using the Glutathione GSH/GSSG Assay kit (Sigma-Aldrich, Schnelldorf, Germany) according to manufacturer’s protocol. In brief, 5% meta-phosphoric acid solution was used to remove protein from samples following centrifuge at 14,000× *g* for 5 min. Supernatant was collected to mix with assay buffer and working reagent for glutathione assay. OD value of each sample and standard at 412 nm at zero and 10 min was measured; then, the results were calculated by the following formula:

GSH_Total_ (µM) = (ΔOD_Sample_ − ΔOD_Blank_) × DF/Slope (µM^−1^)

ΔOD: OD_10min_ − OD_0min_, DF: dilution factor, Slope: standard curve.

### 2.9. Quantitative Real-Time PCR

RNA from colonic tissue was extracted using RNeasy kits (Qiagen, Copenhagen, Denmark), and cDNA synthesis (QuantiTect Reverse Transcription Kit, Qiagen) was performed according to manufacturer’s protocols, as previously described [16]. Primers are listed in Table 1. PerfeCTa SYBR Green FastMIX Low ROX (Quanta Bioscience, Gaithersburg, MD, USA) was used for qPCR using the following program: initial denaturation step at 95 °C for 2 min, followed by 40 cycles of 95 °C for 15 s and 60 °C for 20 s. Relative expression was calculated using the ^ΔΔ^CT method using *Gapdh* as a reference gene.

### 2.10. Statistical Analysis

Statistical analysis was determined by one way ANOVA or *t*-test with Graph Pad Prism (Version 8.0, Grahpad Prism, San Diego, CA, USA). Weight gain and gene expression were analyzed by two-way ANOVA (infection and diet), with *p* value < 0.05 considered significant. Changes in relative distribution of zOTUs were determined with the G-test of independence (based on Bonferroni-corrected *p* value < 0.05), followed by *t*-tests. All data were represented as means ± standard error of mean (SEM).

## 3. Results

### 3.1. PTS-PTSO Decreases Reactive Oxygen Species and Nitric Oxide Production in Macrophages

**Figure 2:**
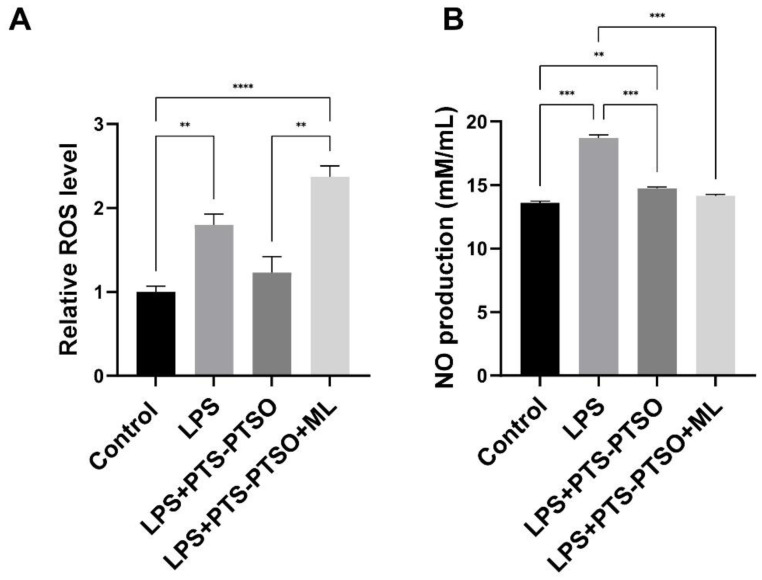
PTS-PTSO inhibits reactive oxygen species (ROS) production and nitric oxide (NO) release in murine macrophages. (**A**) ROS level in RAW264.7 cells stimulated with PTS-PTSO (20 µM) + LPS or PTS-PTSO + LPS combined with Nrf2 inhibitor ML385 (5 µM), n = 6 replicates per treatment. (**B**) NO production in RAW264.7 cells stimulated with PTS-PTSO (20 µM) + LPS or PTS-PTSO + LPS combined with Nrf2 inhibitor ML385 (5 µM), n = 4 replicates per treatment. Data bars represent mean ± SEM. ** *p* ≤ 0.01, *** *p* ≤ 0.001, **** *p* ≤ 0.001.

**Figure 3:**
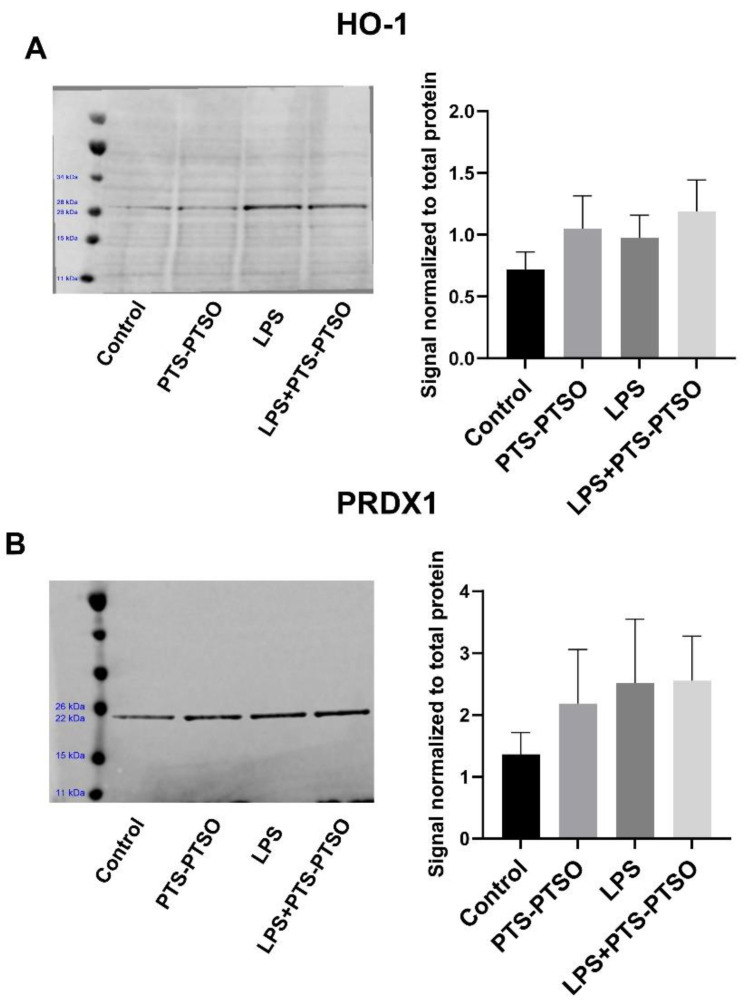
PTS-PTSO does not affect antioxidant protein expression of HO-1 and PRDX1 in murine macrophages. Western blot was conducted to assess HO-1 and PRDX1 protein expression. (**A**) Signal normalized to total protein of HO-1 in RAW264.7 cells stimulated with PTS-PTSO and LPS. n = 6 replicates per treatment group (**B**) Signal normalized to total protein of PRDX1 in RAW264.7 cells stimulated with PTS-PTSO and LPS, n = 8 replicates per treatment.

ROS and NO are involved in many physiological processes, especially oxidative damage, apoptosis and inflammation [18,19,20]. We have previously shown that PTS-PTSO limits inflammatory cytokine production and upregulates genes involved in antioxidant responses in mouse macrophages in vitro [15]. To confirm the functional antioxidant activities of PTS-PTSO, we first stimulated RAW264.7 cells with 20μM PTS-PTSO as well as with LPS, with or without the Nrf2 inhibitor ML385. As shown in Figure 2, LPS significantly increased ROS level and NO production, and PTS-PTSO tended to inhibit ROS release as well as substantially decrease NO levels. Importantly, the Nrf2 inhibitor significantly prevented the PTS-PTSO’s ability to reduce ROS production, indicating that a major mechanism of the antioxidant activity of PTS-PTSO is its functioning as an Nrf2 activator. In contrast, ML385 treatment had no effect on NO levels, suggesting that PTS-PTSO induces different pathways to regulate ROS production and NO release. To explore whether PTS-PTSO also induced the expression of Nrf2-related proteins in RAW264.7 cells, we quantified heme oxygenase 1 (HO-1) and Peroxiredoxin 1 (PRDX1) levels as antioxidant proteins involving in oxidative stress and inflammation in response to ROS [21,22]. An increasing trend was shown in PTS-PTSO-stimulated cells; however, there were no statistical differences in the expression of PRDX1 and HO-1 (Figure 3). Collectively, these data show that PTS-PTSO inhibits the production of LPS-induced oxidative stress in macrophages whilst tending to increase the production of proteins involved in antioxidant responses.

### 3.2. PTS-PTSO Modulates Serum Nitric Oxide and the Faecal Microbiota Composition in Mice

**Figure 4:**
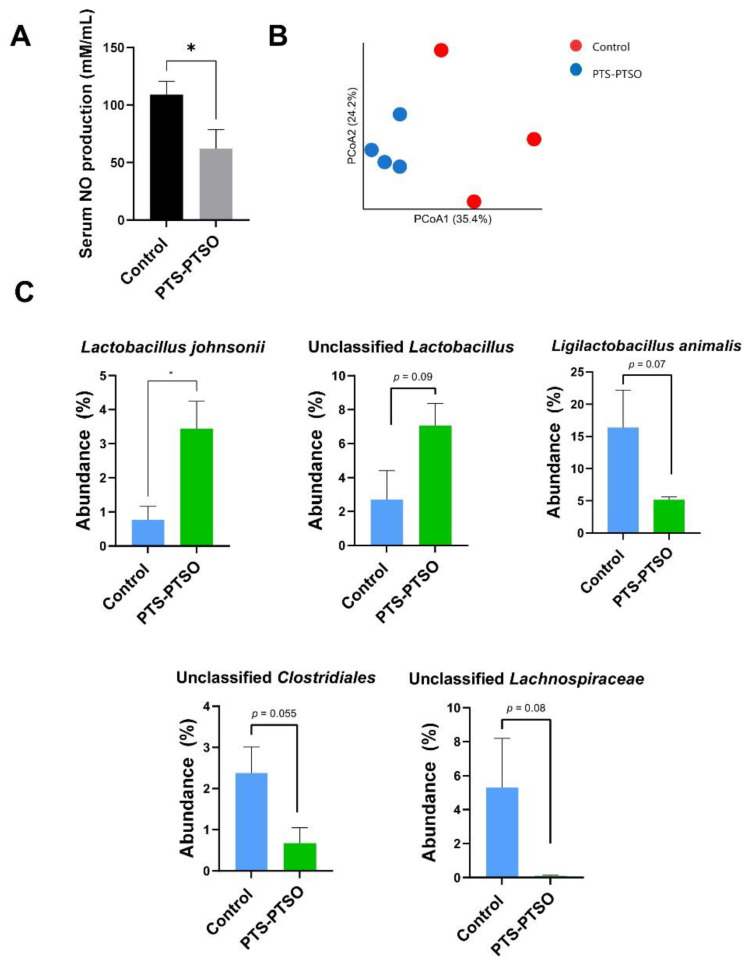
PTS-PTSO modulates serum nitric oxide (NO) levels and faecal microbiota composition. (**A**) Serum nitric oxide level in mice fed with water or 1 mg/kg PTS-PTSO after a 2-week treatment period. (**B**) Principal component analysis based on Bray–Curtis differences in feacal microbiota. (**C**) Relative abundance of bacteria taxa found to differ between PTSO-PTS-treated and control groups. * *p* < 0.05. Data bars represent mean ± SEM. n = 3–4 per group.

Having established that PTS-PTSO exerted significant functional activity in in vitro cellular models, we next explored whether in vivo intake in mice affected oxidative stress responses and the gut microbiota community structure. First, to explore the antioxidant ability of PTS-PTSO in vivo, serum NO levels were analyzed, which revealed that PTS-PTSO intake for 2 weeks significantly decreased NO production (*p* < 0.05), confirming the results of the in vitro assays (Figure 4A). Next, 16S rRNA gene amplicon-based sequencing was conducted to analyze faecal microbial communities to explore the effects of PTS-PTSO on the murine gut microenvironment. Mice were administered PTS-PTSO (1 mg/kg body weight), or vehicle control, in drinking water for 14 days and faecal samples were collected for analysis. The results indicated that PTS-PTSO significantly changed the faecal microbiota composition (*p* = 0.035 by ANOSIM; Figure 4B). PTS-PTSO was associated with a significantly increased abundance of *Lactobacillus johnsonii*, and tended to also increase the abundance of other unclassified members of the *Lactobacillus* genus, (Figure 4C). In contrast, *Ligilactobacillus animalis* tended to decrease in abundance, whilst we also noted a trend of a reduction in members of the *Clostridiales* order and *Lachnospiraceae* family (Figure 4C). Thus, consumption of PTS-PTSO reduced oxidative stress in vivo, and the enrichment of lactobacilli may indicate a modification of the GM towards a composition putatively associated with reduced inflammation.

### 3.3. PTS-PTSO Reduces Faecal Excretion of Citrobacter Rodentium

**Figure 5:**
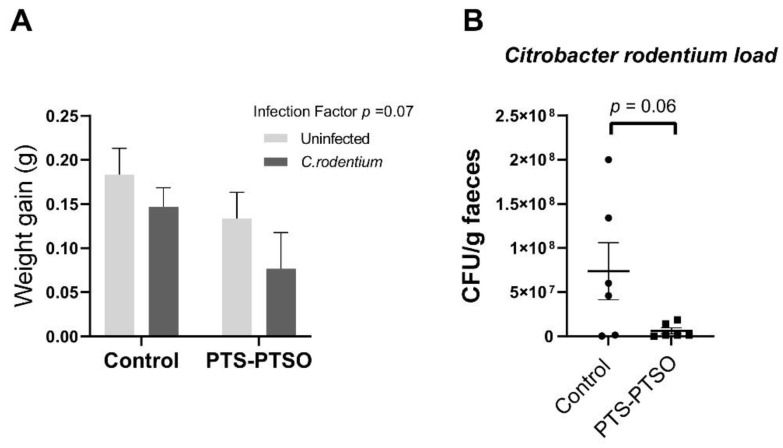
PTS-PTSO reduces *C. rodentium* faecal load. (**A**) Weight gain per day during infection phase in mice given control water, 1 mg/kg PTS-PTSO and 10^9^ CFU *C. rodentium*. (**B**) Faecal load of *C. rodentium* in mice treated with or without PTS-PTSO. Data bars represent mean ± SEM. n = 6 per group.

Given the potentially beneficial effects of PTS-PTSO on the gut environment, we next investigated its effects on enteric pathogen infection. Mice received 1 mg/kg PTS-PTSO (or vehicle control) in drinking water for 7 days prior to infection, and then continued to receive the supplemented water during a 6-day infection period with *C. rodentium*. In parallel, uninfected mice received the same treatments. Compared with uninfected mice, *C. rodentium*-infected mice tended to have a lower growth rate during infection (Figure 5A; *p* = 0.07), and so did the groups dosed with PTS-PTSO (*p* = 0.15). Moreover, PTS-PTSO resulted in a reduction in *C. rodentium* burdens in the faeces (Figure 5B; *p* = 0.06). Thus, PTS-PTSO supplementation tended to reduce *C. rodentium* colonisation.

### 3.4. PTS-PTSO Increases Nrf2-Related Gene Expression in Mouse Colonic Tissue

**Figure 6:**
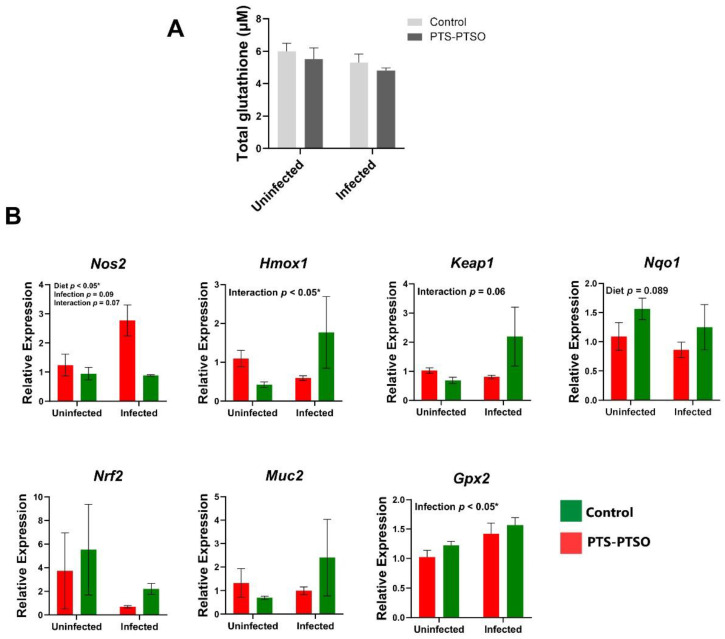
PTS-PTSO induces tendency of localized antioxidant response related to Nrf2 signaling against *C. rodentium*. (**A**) Total glutathione level of serum in naïve or *C. rodentium* infected mice treated with water or 1 mg/kg PTS-PTSO at 6 days p.i. (**B**) Relative gene expression of *Nos2*, *Gpx2*, *Nrf2*, *Nqo1*, *Muc2*, *Hmox1* and *Keap1*. Data bars represent mean ± SEM. n = 6 per group. * *p* < 0.05.

*C. rodentium* and other enteric pathogens are known to increase oxidative stress in the intestine [23,24]. Mice fed diets deficient in the natural antioxidants selenium and vitamin E have increased *C. rodentium* burdens, together with the increased inflammation and expression of oxidative stress proteins, suggesting that the modulation of oxidative stress responses by dietary components may potentially contribute to the lower pathogen burdens [23]. Given the modulatory effects of ROS production and Nrf2 signaling in PTS-PTSO stimulated cells in vitro, we further explored the expression of Nrf2 related genes in vivo in *C. rodentium*-infected mice. At day 6 p.i., the total serum glutathione level was not different in any of the treatment groups (Figure 6A). *Nos2* expression was upregulated in the colon of *C. rodentium* infected mice but significantly restricted by PTS-PTSO intake, as well as in uninfected mice (Figure 6B). There was a significant interaction between diet and infection for the antioxidant genes *Hmox1* and *Keap1,* with PTS-PTSO inducing expression in infected mice that was significantly higher than for the other groups. Similarly, the expression of *Nqo1*, encoding NAD(P)H Quinone Dehydrogenase 1, an enzyme with cytoprotective properties against oxidative stress regulated by Nrf2 pathway [25], was upregulated by PTS-PTSO in both uninfected mice and infected mice. *Gpx2* expression also tended to be elevated in PTS-PTSO treated mice, and was significantly higher in infected mice than uninfected mice (Figure 6B). *Muc2* and *Nrf2* expression were expressed at a relatively high level by PTS-PTSO in infected mice compared to others. Taken together, these data suggest that PTS-PTSO consumption was related to the increased expression of genes downstream Nrf2 signaling in the colon, which was particularly evident during infection with *C. rodentium*, suggesting that intake of these garlic metabolites had a pronounced role in regulating enteric inflammation and infection in this model.

## 4. Discussion

Enteric infections with bacteria, viruses, and parasites are a major cause of morbidity in animals and humans, especially in developing countries, [26,27]. Numerous studies have suggested that gut commensal residents and diet supplementation both promote intestinal health by regulating immune responses and metabolism [28]. For example, PTS-PTSO has been shown to lower levels of enteropathogens in broiler chickens, and the ileal histological structure and productive parameters were improved, demonstrating that PTS-PTSO may be a beneficial alternative treatment option in the control of pathogenic bacteria [29]. Our previous work suggested that PTS-PTSO regulates inflammatory responses in vitro and enteric parasite-induced inflammation in vivo [15]. However, it remains unclear whether PTS-PTSO is effective against intestinal bacterial infection and has an impact on antioxidant activity. Here, we conducted antioxidative assays and a faecal microbiota analysis of PTS-PTSO, showing that PTS-PTSO suppresses *C. rodentium* faecal load in mice, and there is a modulatory effect of PTS-PTSO on Nrf2-signaling-based antioxidant activity both in vitro and during *C. rodentium* colonization in the colon.

ROS mediates many biological functions, such as energy metabolism and excessive ROS can interact with lipid, DNA, proteins to induce oxidative damage, and promote pro-inflammatory signaling activation under stress [30,31]. It is well-known that Nrf2 is a crucial transcription factor against oxidative stress by binding to antioxidant response elements (AREs) to induce antioxidant proteins, and thereby removing ROS from damaged cells [32]. In this study, a significant increase in ROS was observed in LPS-activated cells treated with PTS-PTSO and ML385 compared to LPS-activated cells stimulated with PTS-PTSO only, indicating that PTS-PTSO exerts antioxidant effects by inhibiting intracellular ROS via Nrf2-induced signaling. Meanwhile, NO induced by inducible nitric oxide synthase (iNOS), has been recognized as a main factor in the control of infectious diseases, tumor development and immune responses [33,34]. In our results, the Nrf2 signaling inhibitor ML385 had no effect on NO production mediated by PTS-PTSO, suggesting that PTS-PTSO may target other sites to decrease NO release in LPS-activated macrophages rather than Nrf2 signaling. In contrast, PTS-PTSO had only minor effects on the expression of the antioxidant proteins HO-1 and PRDX1.

Commensal residents in the gut have previously been shown to regulate intestinal mucosa homeostasis and cytokine production against oxidative stress by releasing metabolites such as short-chain fatty acids, which change the microbiota composition towards more beneficial taxa, thereby maintaining metabolic and immune homeostasis, as well as intestinal barrier integrity [35]. The main bacterial phyla in the mouse colon comprise *Firmicutes*, *Bacteroidetes*, *Actinobacteria* and *Proteobacteria* [36]. Here, we found an enrichment of *L. johnsonii* as a result of PTS-PTSO intake, whilst members of the *Clostridiales* order and other unclassified *Lachnospiraceae* family members tended to decrease in abundance. Some lactobacilli are well-known for their beneficial health protective properties, such as by modulating oxidative stress through the downregulation of ROS-forming enzymes [37]. Importantly, *L. johnsonii* has the ability to alleviate pro-inflammatory mediators including NO secretion, which is consistent with the decreased serum NO level in PTS-PTSO treated mice [38,39]. Thus, PTS-PTSO supplement has a beneficial effect in vivo, and it may potentially explain the reduced serum NO.

Our previous study investigated the effects of PTS-PTSO against enteric helminth infections using *T. muris*, and we reported no differences in *T. muris* burdens, although PTS-PTSO altered inflammation-related genes and pathways [15]. In the current study, *C. rodentium* infection seemed to induce lower weight gains, and PTS-PTSO could not mitigate this decrease. Importantly, faecal counts of *C. rodentium* were almost significantly lower in PTS-PTSO treated mice compared to in control mice (*p* = 0.06). We hypothesized that PTS-PTSO supplementation might affect *C. rodentium* infection through Nrf2-related signaling affecting levels of the antioxidant molecule glutathione, as well as by altering the gene expression levels of *Nos2*, *Gpx2*, *Nqo1* and *Keap1*, *Hmox1* or by altering mucus secretion (*Muc2*). Interestingly, we found no difference in total glutathione concentration observed in serum. The total glutathione concentration is a major marker of oxidative stress, made up of reduced and oxidized forms involved in multiple redox balances and xenobiotic metabolism [40]. Nrf2 signaling also plays a role in reducing oxidative damage by regulating genes including *Nos2*, *Gpx2*, *Keap1*, *Nqo1* and *Hmox1* [41]. Interestingly, the decreased expression of nitric oxide synthase gene, *Nos2*, was observed, which may explain the reduced serum NO level. The tendency of up-regulation of the other genes in cells of the gut (*Gpx2*, *Keap1*, *Nqo1* and *Hmox1*) in PTS-PTSO fed mice was consistent with enhanced antioxidant activity seen in the cell study, but this effect might be localized, rather than systemic. Importantly, the activity against *C. rodentium* seemed to correlate with Nrf2 related signaling. However, further experiments are necessary to determine whether this has a causative role in the health-promoting effects of PTS-PTSO. Overall, PTS-PTSO may hold potential as a functional food component against infectious pathogens, but we cannot conclude whether the underlying mechanisms are related only to the antioxidant responses or include changes in the mucus layer as well as in the gut microbiota. Further attention will also be important to determine the most effective means of reaching the levels of PTS-PTSO intake that are necessary to show biological effects. Whilst a moderately high level of garlic consumption in humans has been shown to result in PTS intake approaching 0.1 mg/kg body weight [42], this is still somewhat lower than the dosages used in our current experiments. Thus, it may be more feasible to use dietary supplementation with purified forms of PTS-PTSO, as is increasingly being performed, with some success, in livestock production [43].

## 5. Conclusions

PTS-PTSO show potential for reducing anti-oxidative stress in the gut through Nrf2 signaling, and, thus, the regulation of mediators of ROS and NO release, as well as commensal microbiota composition. In addition, PTS-PTSO potentially develop anti-intestinal bacteria ability, which is likely related to Nrf2 signaling. These results provide further insights into PTS-PTSO and related molecules as a supplement that may benefit gut health.

## Figures and Tables

**Table 1:** Primers used for qPCR.

Genes	GeneBank Accession	Primer Sequence (5-3 Forward/Reverse)
*Nrf2*	AH006764.2	F: CGAGATATACGCAGGAGAGGTAAGA R: GCTCGACAATGTTCTCCAGCTT
*Keap1*	AB020063.1	F: CAACTTCGCGGAGCAGATCG R: AGCTGGCAGTGTGACAGGTT
*Nqo1*	NM_008706.5	F: CATCCTGCGTTTCTGTGGCT R: TCTCCTCCCAGACGGTTTCC
*Gpx2*	U62658.1	F: CAAGTATGTCCGACCTGGGG R: GGGTAGGGCAGCTTGTCTTT
*Hmox1*	NM_010442.2	F: GAACCCAGTCTATGCCCCAC R: GCGTGCAAGGGATGATTTCC
*Muc2*	NM_023566.4	F: GTCCTGACCAAGAGCGAACA R: TTGAAGGCCACCACGTTCTT
*Nos2*	NM_010927.4	F: GGTGAAGGGACTGAGCTGTT R: TGCACTTCTGCTCCAAATCCA
*Gapdh*	BC023196.2	F: TATGTCGTGGAGTCTACTGGT R: GAGTTGTCATATTTCTCGTGG

## Data Availability

16S rRNA sequence data is available at SRA (https://www.ncbi.nlm.nih.gov/sra, last accessed on 13 October 2022) under the accession number SUB12140517. All other data is contained within the article.
